# Reshaping the Binding Pocket of the Neurotransmitter:Solute Symporter (NSS) Family Transporter SLC6A14 (ATB^0,+^) Selectively Reduces Access for Cationic Amino Acids and Derivatives

**DOI:** 10.3390/biom12101404

**Published:** 2022-10-01

**Authors:** Catriona M. H. Anderson, Noel Edwards, Andrew K. Watson, Mike Althaus, David T. Thwaites

**Affiliations:** 1 School of Natural & Environmental Sciences, Faculty of Science, Engineering & Agriculture, Newcastle University, Newcastle upon Tyne NE1 7RU, UK; 2 Biosciences Institute, Faculty of Medical Sciences, Framlington Place, Newcastle University, Newcastle upon Tyne NE2 4HH, UK; 3 Department of Natural Sciences & Institute for Functional Gene Analytics, Bonn-Rhein-Sieg University of Applied Sciences, 53359 Rheinbach, Germany

**Keywords:** amino acid transporter, solute carrier, SLC, membrane transport, SLC6A14, SLC6, ATB^0,+^, NSS family, APC superfamily, LeuT

## Abstract

SLC6A14 (ATB^0,+^) is unique among SLC proteins in its ability to transport 18 of the 20 proteinogenic (dipolar and cationic) amino acids and naturally occurring and synthetic analogues (including anti-viral prodrugs and nitric oxide synthase (NOS) inhibitors). SLC6A14 mediates amino acid uptake in multiple cell types where increased expression is associated with pathophysiological conditions including some cancers. Here, we investigated how a key position within the core LeuT-fold structure of SLC6A14 influences substrate specificity. Homology modelling and sequence analysis identified the transmembrane domain 3 residue V128 as equivalent to a position known to influence substrate specificity in distantly related SLC36 and SLC38 amino acid transporters. SLC6A14, with and without V128 mutations, was heterologously expressed and function determined by radiotracer solute uptake and electrophysiological measurement of transporter-associated current. Substituting the amino acid residue occupying the SLC6A14 128 position modified the binding pocket environment and selectively disrupted transport of cationic (but not dipolar) amino acids and related NOS inhibitors. By understanding the molecular basis of amino acid transporter substrate specificity we can improve knowledge of how this multi-functional transporter can be targeted and how the LeuT-fold facilitates such diversity in function among the SLC6 family and other SLC amino acid transporters.

## 1. Introduction

In mammals, transport of solutes such as amino acids, monosaccharides, micronutrients, water-soluble vitamins, minerals, fatty acids, metabolites, and anionic and cationic electrolytes, across cell membranes is mediated by members of the Solute-Linked Carrier (SLC) superfamily of transporters [1,2]. The mammalian SLC superfamily consists of more than 400 transporters divided into 66 SLC families. Members are not categorised within a particular SLC family based upon any single or unifying functional characteristic but rather >20% identity in their primary protein sequences [1].

In eukaryotes, some amino acids can be generated within cells whereas others, the so called essential amino acids, must be acquired from diet as animals have lost the capacity to synthesise those amino acids at levels required to meet demand. Amino acid transporters are, thus, widespread in nature and critical for cellular accumulation of key solutes for essential cellular processes. 

Within the SLC superfamily, more than 60 amino acid transporters are distributed across the following mammalian SLC families; SLC1, SLC6, SLC7, SLC16, SLC17, SLC25, SLC32, SLC36, SLC38, SLC43 and SLC66 [1,2]. The SLC6 family consists of twenty-one members (including two pseudogenes) in the human genome [3]. Although members of the SLC6 family show high levels of sequence identity, function varies within the family including transporters of: neurotransmitters (the biogenic amines norepinephrine, dopamine and serotonin, and the amino acids GABA and glycine); both essential and non-essential proteinogenic amino acids; osmolytes (taurine and betaine) and creatine [3]. SLC6 transporters function as Na^+^-coupled solute cotransporters with the direction of transport being driven by the prevailing transmembrane Na^+^-electrochemical gradient. In the Transport Classification DataBase (TCDB) [4], which includes eukaryote and prokaryote transporters from all kingdoms of life, SLC6 family members are classified within the larger Neurotransmitter:Sodium Symporter (NSS) family, which has the TCDB family identifier 2.A.22 [3,4,5]. 

One such mammalian SLC6 amino acid transporter is commonly known as ATB^0,+^ or SLC6A14 [6]. ATB^0,+^, so called, because it is an Amino acid Transporter with Broad substrate specificity, transporting 18 of the 20 proteinogenic amino acids including both neutral/dipolar amino acids (hence the superscript ^0^) and cationic/dibasic amino acids (hence the superscript ^+^) [6,7,8]. The transporter is also known as SLC6A14 as it is the 14th member of the SLC6 family [3,6]. Human SLC6A14 has the TCDB identifier 2.A.22.2.3. 

Functionally, SLC6A14 is a highly unusual transporter as: it has the broadest substrate specificity of any mammalian amino acid transporter yet characterised; it transports all nine of the essential amino acids; it accepts non-α-amino acids such as β-alanine with low affinity; it accepts some d- as well as l-amino acids; it is both Na^+^ and Cl^−^-dependent [6,8,9,10,11,12,13,14]. SLC6A14 is an electrogenic cotransporter with a Na^+^:Cl^−^:amino acid stoichiometry of 2:1:1 demonstrating that the transporter is driven by electrochemical gradients for Na^+^ and Cl^−^ and membrane potential. It is considered that the transporter concentrates its substrates up to 1000 times inside cells [6] meaning that it should function exclusively as an influx mechanism. The broad substrate specificity for naturally occurring amino acids is reflected in the ability of SLC6A14 to also transport amino acid-based nitric oxide synthase (NOS) inhibitors [15], and amino acid-based prodrugs such as the anti-virals valganciclovir [16] and valacyclovir [17]. In addition, derivatives of the large, aromatic amino acid tryptophan act as non-transported inhibitors, blocking the transporter and preventing transport of other amino acids [18]. 

Prior to identification at the molecular level, the SLC6A14 or ATB^0,+^ transporter was originally identified functionally in two diverse tissues. Firstly, it was characterised at the apical surface of the rabbit ileum and named the β-alanine carrier after one of the key substrates investigated, although it was evident even in those early studies that the transporter was also able to transport both dipolar and cationic α-amino acids [9,19,20,21]. Secondly, it was described as system B^0,+^ in mouse blastocysts, as a broad scope transporter of dibasic and dipolar amino acids, where it transports amino acids across the apical membrane of the trophectoderm [7,22]. At the molecular level, it was originally cloned from human mammary gland and named ATB^0,+^ due to the similar functional characteristics of the cloned transporter to the blastocyst system B^0,+^ [8]. Following expression of SLC6A14 in *Xenopus laevis* oocytes, we have previously demonstrated conclusively that this solute carrier has all the functional characteristics of both the intestinal β-alanine carrier and the blastocyst system B^0,+^ confirming that they represent function of a single transport system [14]. 

Therefore, two known physiological roles for SLC6A14 are, firstly, the supply of amino acids across the luminal surface in distal intestine (most expression studies suggest relatively weak expression in the ileum with stronger colonic expression and luminal surface localisation) [15,17,23,24,25] where SLC6A14 knockout leads to a 75% reduction in colonic uptake of arginine [25], and, secondly, in supply of amino acids to developing blastocysts [7,22]. SLC6A14 has a fairly limited tissue distribution but is also expressed in lung, trachea, retina, mammary tissue and salivary glands [8,17,24]. The third key physiological role identified to date is in respiratory tissues where SLC6A14 is localised at the apical membranes of airway cells [24]. SLC6A14 transports amino acids such as arginine from the airway surface liquid into airway epithelial cells [26] where its amino acid transport function is considered to result in a reduction in attachment of *Pseudomonas aeruginosa* to airway cells [27]. In SLC6A14 knockout mice, or primary human respiratory cell cultures in which SLC6A14 function is inhibited, *P. aeruginosa* attachment to airway tissues is increased [27]. 

A pathophysiological role for SLC6A14 has been proposed in cancer [6]. Due to their rapid rate of cell division, cancer cells have a high demand for uptake of extracellular amino acid substrates as building blocks to feed growth and proliferation. Cancer cells accomplish this by selectively increasing expression of certain plasma membrane amino acid transporters including SLC6A14 [6]. Its ability to mediate ion-driven accumulative transport of 18 of the 20 proteinogenic amino acids makes SLC6A14 an ideal candidate transporter for upregulation in cancer and increased SLC6A14 expression has been observed in colorectal cancer [28], cervical cancer [29], estrogen receptor positive breast cancer [30] and pancreatic cancer [31]. The role of SLC6A14 in supply of amino acids to cancer cells appears confirmed as SLC6A14 knockout leads to a decrease in tumour growth in mouse models of estrogen receptor positive breast cancer [32] and pancreatic cancer [33]. SLC6A14 has potential as a therapeutic target for both drug delivery and treatment of cancer [6], but also in other pathophysiological conditions associated with increased expression of this transporter such as the inflammatory bowel diseases ulcerative colitis and Crohn’s disease [6,34,35]. In cystic fibrosis, SLC6A14-mediated arginine uptake has been shown to modulate F508del-CFTR function via a nitric oxide-mediated pathway [36].

The multifaceted physiological and pathophysiological roles of SLC6A14 reflect its unique ability, among human solute carriers, to transport a structurally diverse array of naturally occurring amino acids, derivatives and therapeutic agents including cationic solutes. The structural characteristics underlying the functional promiscuity in the NSS transporter SLC6A14 remain unknown. We have previously identified a residue position in transmembrane domain (TM) 3 in the Amino Acid/Auxin Permease (AAAP) family (2.A.18) amino acid transporters SLC36A2 (PAT2) and SLC38A5 (SNAT5) which determines the space available for an amino acid substrate side chain within the transporter binding pocket and thus influences substrate specificity [37]. In SLC36A2, mutating the TM3 residue F159 to F159I, F159T or F159C sequentially increased the length of substrate side chain that could access the binding pocket and consequently expanded the substrate specificity of SLC36A2 to include larger, dipolar amino acids [37]. In contrast, mutation of A138 in TM3 of SLC38A5 to A138T or A138I had the opposite effect, decreasing available space within the binding pocket leading to a change of the substrate specificity to favour amino acids with smaller side chains [37]. The objective of this investigation was to determine whether the equivalent position in TM3 of SLC6A14 also modulates substrate specificity in this NSS family transporter. Here, we describe how altering the amino acid residue occupying that equivalent position in TM3 of SLC6A14 selectively alters transport of cationic amino acids and related NOS inhibitors by SLC6A14.

## 2. Materials and Methods

### 2.1. Materials

[^3^H] and [^14^C] radiochemicals were from PerkinElmer (Seer Green, UK) except for [^14^C]alanine from Hartmann Analytic (Braunschweig, Germany) and [^3^H]serine from American Radiolabeled Chemicals (St. Louis, MO, USA). Site-directed mutagenesis QuikChange Lightning kit, reagents and primer design were from Agilent Technologies (Stockport, UK). NOS inhibitors and related amino acid derivatives were from SigmaAldrich (Gillingham, UK), Santa Cruz (Heidelberg, Germany) and Enzo Life Sciences (Exeter, UK).

### 2.2. Site-Directed Mutagenesis and Expression in Xenopus Laevis Oocytes

Human SLC6A14 in the pSPORT1 plasmid was a gift from V. Ganapathy (Texas Tech University) [38]. Site-directed mutagenesis was carried out according to the manufacturer’s instructions. The PCR parameters used were: 2 min at 95 °C; 18 cycles of 20 s at 95 °C (denaturation), 10 s at 68 °C (annealing), 30 s/kb at 68 °C (extension); 5 min at 68 °C. Mutations were verified by sequencing of the entire open reading frame (GATC Biotech, London, UK). cRNA was produced by in vitro transcription using the Invitrogen mMessage mMachine T7 Ultra kit (Fisher Scientific, Loughborough, UK). Female *Xenopus laevis* were obtained from Xenopus1 (Dexter, MI, USA) and handled in accordance with UK Home Office Schedule 1 procedures. Alternatively, *X. laevis* ovaries were purchased from the European *Xenopus* Resource Centre (Portsmouth, UK). Oocytes were prepared and injected with 50 nl cRNA (1 mg/mL) or water (control), as described previously [14,37,39,40,41,42]. Oocytes were incubated at 16–18 °C in Barth’s solution for 2–3 days until required.

### 2.3. Amino Acid Transport Assay

Uptake of radiolabelled amino acids (1–5 µCi/mL) was measured over 40 min at room temperature (20 °C) in uptake solution (100 mM NaCl, 2 mM KCl, 1 mM CaCl_2_, 1 mM MgCl_2_, 10 mM HEPES, adjusted to pH 7.4 with Tris base), as described previously [14,39,40,41,42]. Uptake into water-injected oocytes was always determined, under identical conditions, as a control. 

### 2.4. Two-Electrode Voltage Clamp (TEVC)

Electrophysiology was used to measure rheogenic inward current associated with Na^+^/Cl^−^/amino acid-cotransport via SLC6A14 [14]. Oocytes were placed in a Lucite chamber and perfused, at room temperature, with uptake solution via a gravity-driven perfusion system. Chlorided silver wires served as recording electrodes. Reference electrodes were connected to the chamber using 1 M KCl agar (3%) bridges. Intracellular microelectrodes (1–10 MΩ resistance) were pulled from borosilicate glass capillaries and filled with 1 M KCl. The membrane potential (V_M_) was clamped to –60 mV, with a TEVC amplifier (Warner Instruments, Hamden, CT, USA). Transmembrane currents (I_M_) were low-pass filtered at 1 kHz (LPF-202, Warner Instruments) and recorded by a strip-chart recorder. Current traces were digitised using Inkscape (version 1.0.2-2). The current induced by various amino acids was calculated as the difference between I_M_ before amino acid exposure (baseline) and I_M_ 60 or 120 s into amino acid exposure. SLC6A14-specific current was calculated by subtracting current in water-injected (control) oocytes measured under identical conditions.

### 2.5. Phylogeny

Phylogenetic relationships between SLCs were examined, essentially as described previously [37]. Human sequences for all members of SLC5, SLC6, SLC7, SLC11, SLC12, SLC32, SLC36, and SLC38 were retrieved via the Bioparadigms database [1]. Additional, non-human, sequences for the NSS family tree were retrieved from the TCDB [4]. Alignments were done using MUSCLE (default parameters) [43] and trimmed using TrimAl v1.4 [44], with trimming parameters defined by the automated1 option. Phylogenies were inferred in PhyloBayes [45] using the CAT60 or CAT20 models [46], with chain convergence confirmed by bpcomp and tracecomp. Sequence alignments (trimmed and untrimmed) associated with the phylogenies are available at figshare using the link: 10.6084/m9.figshare.20182088.

### 2.6. Alignment and Homology Modelling

HHPred [47] and Modeller [48] were used (default settings, http://www.toolkit/tueingen/mpg.de) [49] to create alignments and subsequent models of human SLC6A14 based on NSS family proteins with resolved structures. These structures were: LeuT from *Aquifex aeolicus* (protein data bank [PDB] ID 2A65, with leucine substrate-bound) [50], human SLC6A19 (6M17, with leucine substrate) [51], human SLC6A9 (6ZBV, with the benzoylisoindoline inhibitor Cmpd1) [52], dopamine transporter (dDAT) from *Drosophila melanogaster* (4XP9, with d-amphetamine substrate) [53], and human SLC6A4 (5I6X, with paroxetine inhibitor) [54], all with HHPred probability scores of 100%. Homology models of SLC6A14 were superimposed on crystal structures and visualised using PyMol (2.1.0, open source, Schrödinger, LLC). Predicted structures for human SLC6A14 and SLC6A9 were also taken from the AlphaFold protein structure database [55,56]. A full-length protein sequence alignment of SLC6 family transporters and LeuT was created using MUSCLE [43].

### 2.7. Data and Statistical Analysis

Transport data are mean ± SEM and are typically expressed as pmol.oocyte^−1^.[duration]^−1^. Calculation of Michaelis-Menten kinetics and statistical analyses were carried out using GraphPad Prism 9. Two-way ANOVA with Tukey’s or Sidak’s multiple comparisons post-tests or two-tailed, unpaired t-tests were used to compare mean values, as appropriate. Figures were prepared with Prism 9 and Inkscape 1.0.2-2. 

## 3. Results

**Figure 1:**
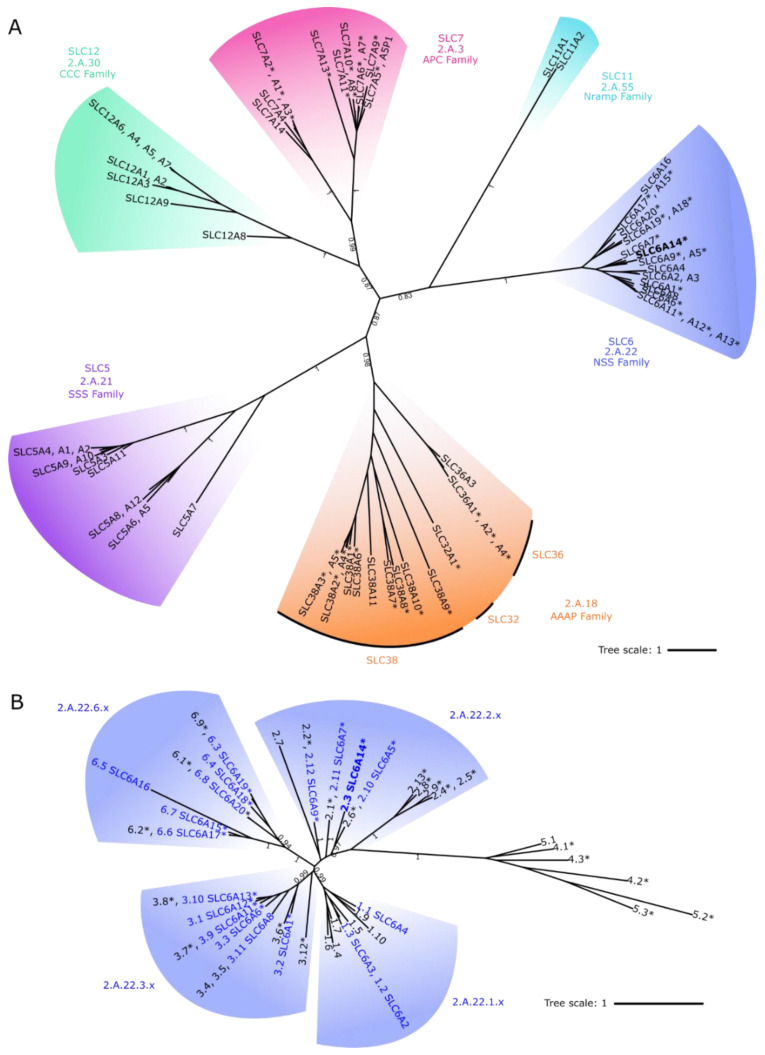
Amino acid transporters are found within multiple families of the APC superfamily including the Neurotransmitter:Sodium Symporter (NSS, 2.A.22) family. (**A**) Phylogenetic analysis of human APC superfamily proteins predicted to have a LeuT-fold core structural motif despite transporting many different solute types. Asterisks: proteins known to transport at least one type of amino acid. SLC families are as follows: SLC5 (in the Solute:Sodium Symporter (SSS) Family, 2.A.21), SLC6 (in the Neurotransmitter:Sodium Symporter (NSS) Family, 2.A.22), SLC7 (in the Amino Acid-Polyamine-Organocation (APC) Family, 2.A.3), SLC11 (in the Metal Ion (Mn^2+^-iron, Nramp) Transporter Family, 2.A.55), SLC12 (in the Cation-Chloride Cotransporter (CCC) Family, 2.A.30), and SLC32, SLC36, & SLC38 (in the Amino Acid/Auxin Permease (AAAP) Family, 2.A.18). The tree was inferred using Phylobayes [45] and the C60 model [46]. Where necessary, labels are grouped to prevent overlap. Branch values: posterior probabilities, indicative of the level of support, shown for key internal branches with a value > 0.8. Supplemental Figure S1A shows the full tree with all support values and labels. (**B**) Phylogenetic analysis of the NSS (2.A.22) family using eukaryote and prokaryote sequences assigned to this family on TCDB. Proteins are labelled by the end of their TCDB identifier and, for human proteins (blue text), their SLC gene name. Where necessary, labels are grouped to prevent overlap. Asterisks: proteins known to transport at least one type of amino acid. All subfamily clades include amino acid transporters except the 2.A.22.1 subfamily of monoamine transporters (SLC6A2, NET; SLC6A3, DAT; SLC6A4, SERT). The tree was inferred using Phylobayes [45] and C20 model [46]. Levels of support are shown for key internal branches with a value > 0.8. Supplemental Figure S1B shows the full tree, all support values and labels.

SLC6A14, and the wider NSS family, form part of the largest grouping of amino acid transporters across all kingdoms of life within the Amino acid-Polyamine-organoCation (APC) superfamily, as classified by the Transporter Classification DataBase (TCDB) [4,5]. Most transporters within that APC superfamily are considered to possess a similar structural fold, known commonly as the LeuT-fold after the first amino acid transporter within that superfamily to have its structure resolved [50]. The LeuT-fold consists of a 5 + 5 transmembrane spanning domain inverted structural repeat [50]. Figure 1A and Supplemental Figure S1A show human LeuT-fold solute carriers which, alongside SLC6 (from the NSS family, 2.A.22), includes the following solute carrier families: SLC5 (in the SSS family, 2.A.21), SLC7 (in the APC family, 2.A.3), SLC11 (in the Nramp family, 2.A.55), SLC12 (in the CCC family, 2.A.30), and SLC32, SLC36 and SLC38 (in the AAAP family, 2.A.18) [5,37]. Nearly 60% of all the functionally characterised solute carriers shown in Figure 1A are known to transport one or more amino acids (indicated with asterisks), although they have vastly differing substrate specificity and modes of transport. These mammalian APC superfamily amino acid transporters are found within the SLC6, SLC7, SLC32, SLC36 and SLC38 families [5,37]. Other LeuT-fold human solute carriers can transport monosaccharides, vitamins, monocarboxylates, organic cations or inorganic ions (all within SLC5), biogenic amines (SLC6), divalent metals (SLC11), or inorganic anions and cations (SLC12) [1,2]. Thus, the LeuT-fold has allowed evolution of great flexibility in substrate specificity but does appear to be particularly efficient at enabling transmembrane transport of amino acids [37].

Figure 1B and Supplemental Figure S1B suggest that, within the NSS family, the SLC6 transporters are grouped into four subfamilies [3] and are distantly related to LeuT (2.A.22.4.2) [50] and other prokaryote NSS transport proteins including the broad scope transporter MhsT (2.A.22.5.3) [57], and the selective tryptophan and tyrosine transporters TnaT (2.A.22.4.1) [58] and Tyt1 (2.A.22.5.2) [59]. Of the four mammalian SLC6 subfamilies, one is specific for neurotransmitter monoamines (2.A.22.1.x) with the other three including multiple amino acid transporters (Figure 1A,B). These SLC6 amino acid transporters (indicated with asterisks) have highly varied substrate selectivity, some being specific for a single amino acid (e.g., glycine), others transporting a range of different proteinogenic dipolar amino acids, whereas others selectively transport related solutes such as GABA, betaine or taurine [3]. SLC6A14 (2.A.22.2.3) is grouped (Figure 1B) with three transporters of restricted substrate specificity: the proline transporter SLC6A7 (PROT, 2.A.22.2.11) and the glycine transporters SLC6A9 (GlyT1, 2.A.22.2.12) and SLC6A5 (GlyT2, 2.A.22.2.10) [3]. SLC6A14 is unique, within SLC6, in being able to transport cationic as well as all dipolar proteinogenic amino acids.

**Figure 2:**
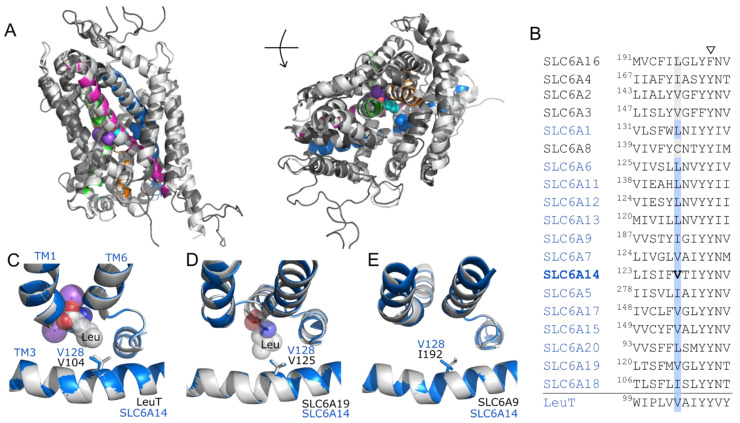
V128 occupies a key position, towards the bottom of the binding pocket, in the broad-spectrum amino acid transporter SLC6A14; the residue at this position varies within the SLC6 family. (**A**) Homology model of SLC6A14 (2.A.22.2.3, dark grey and coloured helices) superimposed on the prokaryote LeuT transporter (2.A.22.4.2) crystal structure (2A65, outward-occluded, leucine-bound, light grey helices) from which it was derived [50]. The key TM domains predicted to be involved in substrate binding in many LeuT fold structures are TM1 (green), TM3 (blue), TM6 (orange), TM8 (magenta). Spheres: residue V128 in TM3 of SLC6A14 (blue), leucine substrate (cyan), Na^+^ ions (purple), both bound within the LeuT binding pocket. A section of the large extracellular loop between TM3 and TM4 is omitted. Left-hand image: protein “side on”, as it would reside in the membrane. Right-hand image: “top-down”, above the plane of the membrane, looking down into the binding pocket. (**B**) Alignment of the central section of TM3 in all human SLC6 proteins and the bacterial homologue LeuT. The pseudogenes SLC6A10P and SLC6A21 are excluded. Alignment was created with full-length protein sequences using MUSCLE. The residue equivalent to V104 in LeuT (V128 in SLC6A14) is highlighted in blue for amino acid transporting proteins and in grey for those which are not known to transport amino acids (i.e., the monoamine transporters and the orphan transporter SLC6A16). Arrowhead indicates the highly conserved residue position equivalent to the gating residue Y163 in LeuT [50]. (**C**–**E**) Homology models of SLC6A14 (blue) created using LeuT (2A65, leucine-bound, outward occluded), human SLC6A19 (6M17, leucine-bound, outward-occluded) or human SLC6A9 (6ZBV, Cmpd1 inhibitor-bound, inward-open) crystal structures (grey), respectively. Only the central sections of TM3, TM1 and TM6 are shown. Sticks: V128 in each SLC6A14 model (blue) and equivalent residue in the resolved structure (grey). Spheres: leucine (grey/blue/red), Na^+^ (purple) bound within the crystal structures of LeuT and SLC6A19. N.B. The large benzoylindoline inhibitor, Cmpd1, which locks SLC6A9 in an inward confirmation, has been omitted for clarity. Additional homology models are shown in Supplemental Figure S2.

Little is known about the structural basis of SLC6A14′s broad substrate specificity and how it can transport cationic as well as dipolar amino acids. Previously, we have identified a residue in TM3 of AAAP amino acid transporters (Figure 1A and Figure 2) which, when changed, can determine which amino acids are accepted as substrates and their relative affinity [37,60]. This residue position is equivalent to position V104 in TM3 of LeuT which is known to line the bottom of the binding pocket [50,61]. The SLC6A14 residue, equivalent to V104 in LeuT, was identified by creating structural homology models and by multi-sequence alignment of the SLC6 family (Figure 2A and Figure S2). Both methods identified SLC6A14 V128 as being in the equivalent position in the central section of TM3 (Figure 2A–C). Like LeuT V104, SLC6A14 V128 sits towards the bottom of the binding pocket which was also observed in the AlphaFold prediction of the SLC6A14 structure (Supplemental Figure S2). Crystal structures have recently been published for two human SLC6 amino acid transporters: SLC6A9 (GlyT1) [52] and SLC6A19 (B^0^AT1) [51]. Each of these structures was used to create SLC6A14 homology models which were then overlaid on the respective crystal structures (Figure 2D,E). Analysis of residue positioning in TM3 in each model, confirmed the alignment results in Figure 2B and shows SLC6A14 V128 to be equivalent to V125 in SLC6A19 and I192 in SLC6A9 (Figure 2D,E). For clarity, only sections of TM1, TM3 and TM6 are shown in Figure 2C–E. Analysis of the TM3 residue position in all human SLC6 transporters (Figure 2B and Figure S2) shows that it varies, in both amino acid-transporting and monoamine-transporting SLC6 transporters, being mostly Val, Ile or Leu. However, there is no obvious correlation between the size of the residue side chain (Figure 2) occupying that position and the known substrate specificity of each transporter. This contrasts with observations in the AAAP transporters SLC36A2, SLC38A5 and the related insect transporters CG1139 (from *Drosophila melanogaster*) and ApNEAAT1 (from *Acyrthosiphon pisum*) [37,60].

**Figure 3:**
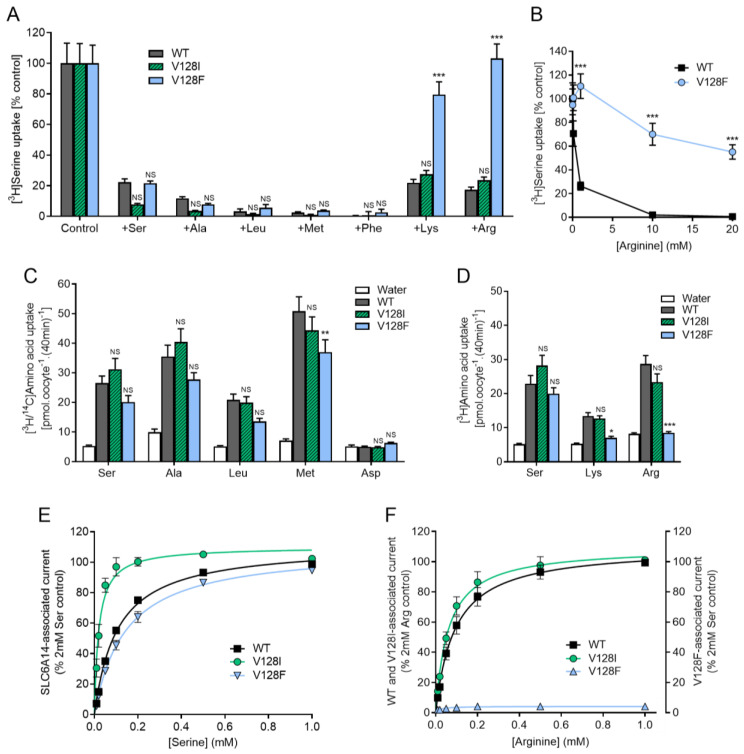
Substitution of V128 in TM3 selectively reduces transport of the dibasic amino acids lysine and arginine via SLC6A14. (**A**,**B**) Serine (10 µM) uptake into oocytes expressing SLC6A14 wild-type (WT) transporter, or SLC6A14 with the mutations V128I or V128F. Uptake was measured in the absence (control) and presence of unlabelled amino acids [(**A**) 2 mM; (**B**) 0–20 mM]. Transporter-specific uptake is shown (after the subtraction of background uptake measured in water-injected oocytes under identical conditions). n = 18–20. ***, *p* < 0.001; NS, *p* > 0.05 vs. WT. (**C**,**D**) Uptake of various radiolabelled amino acids (all 10 µM). Oocytes injected with water, rather than cRNA, were used as a control to determine background endogenous amino acid uptake. n = 19–20. ***, *p* < 0.001; **, *p* < 0.01; *, *p* < 0.05; NS, *p* > 0.05 vs. WT. (**E**,**F**) SLC6A14-associated inward current measured by TEVC. Current was measured 60 s after sequential addition of increasing concentrations (0.01–1 mM) of serine (**E**) or arginine (**F**). Current measured prior to amino acid addition (i.e., in the superfusion solution but absence of amino acid) was subtracted to give amino acid-associated current. Mean current in water-injected oocytes was subtracted to give SLC6A14-specific current. Data were plotted as % of current induced by 2 mM serine or arginine. n = 6, (**E**); n = 3, (**F**). See Supplemental Figure S3 for example traces. Curves are fitted to Michaelis-Menten kinetics [r^2^ = 0.989, WT; 0.839 V128I; 0.976 V128F in (**E**). r^2^ = 0.968 WT; 0.959 V128I; N.D. V128F in (**F**)].

To test whether this residue position (SLC6A14 V128) in TM3 could influence the extremely broad range of amino acids accepted by the SLC6A14 binding pocket, this residue was mutated to both the larger, more hydrophobic Ile (V128I) and the even bulkier aromatic residue Phe (V128F). Previous work showed that F159 in SLC36A2 limits that transporter to small dipolar amino acids such as glycine, alanine and proline [37]. Functional analysis of both SLC6A14 mutants is shown in Figure 3.

Both SLC6A14 mutants V128I and V128F were functional and elicited significant (*p* < 0.001) [^3^H]serine uptake above that measured in water-injected control oocytes (Figure 3). The degree of inhibition of serine (10 µM) uptake in the presence of excess (2 mM) unlabelled competitor amino acids showed no significant difference (*p* > 0.05) between SLC6A14-V128I and the wild-type transporter. However, there was a small, but insignificant, increase in the inhibition caused by alanine (from 88.2 ± 1.1 to 96.5 ± 0.4 %) and (unlabelled) serine (from 77.6 ± 2.1 to 94.2 ± 0.7 %) (both 2 mM) suggesting that the affinity for small amino acids may have increased in the V128I mutant presumably due to an improvement in fit of the substrate within the mutated binding pocket (Figure 3A). 

In the SLC6A14-V128F mutant, there was no change in the degree of inhibition caused by dipolar amino acids of varying size (serine, alanine, leucine, methionine, phenylalanine) compared to wild-type SLC6A14. In contrast, in SLC6A14-V128F there was a large and significant (*p* < 0.001) reduction in competitive inhibition by the longer side-chain containing, cationic amino acids lysine and arginine. Figure 3B shows that the affinity for arginine binding in SLC6A14 decreased from a K_i_ of << 1 mM in the wild-type transporter to a K_i_ of > 20 mM in the V128F mutant. 

The uptake of different amino acids (all 10 µM) was measured in wild type SLC6A14 and both V128I and V128F mutants (Figure 3C,D). There was no significant change (*p* > 0.05) in uptake of any amino acid in V128I compared to wild type. However, uptake of lysine and arginine was reduced in SLC6A14-V128F to the point that it was no different (*p* > 0.05) to that measured in water-injected control oocytes (Figure 3D).

Rheogenic transport via SLC6A14 was determined by two-electrode voltage clamp measurements (Figure 3E,F). Increasing concentrations of serine (Figure 3E) or arginine (Figure 3F) were added to the solution bathing each voltage-clamped oocyte and the current elicited after 1 min was used to plot Michaelis-Menten kinetics to allow estimation of affinity (K_m_) (example traces are shown in Supplemental Figure S3). In SLC6A14-V128I, the affinity for serine increased by five-fold from a K_m_ of 106 ± 5 µM in wild-type SLC6A14 to a K_m_ of 20 ± 3 µM (*p* < 0.001, one-way ANOVA). There was a small but insignificant change in the affinity for arginine (K_m_ = 91 ± 11 µM for wild-type SLC6A14 versus K_m_ = 60 ± 8 µM for SLC6A14-V128I, *p* = 0.0783, two-tailed, unpaired t-test). The capacities of the serine and arginine currents are not shown in Figure 3 but were calculated as: serine, V_max_ = 233 ± 13 µA for wild-type versus V_max_ = 123 ± 26 µA for SLC6A14-V128I; arginine, V_max_ = 232 ± 19 µA for wild-type versus V_max_ = 214 ± 17 µA for SLC6A14-V128I. In SLC6A14-V128F, the affinity for serine was reduced to 142 ± 11 µM (*p* < 0.01, Figure 3E) and the V_max_ was calculated as 136 ± 12 µA. In stark contrast, SLC6A14-V128F had no discernable affinity for arginine, with no arginine-associated currents being detected (Supplemental Figure S3). 

**Figure 4:**
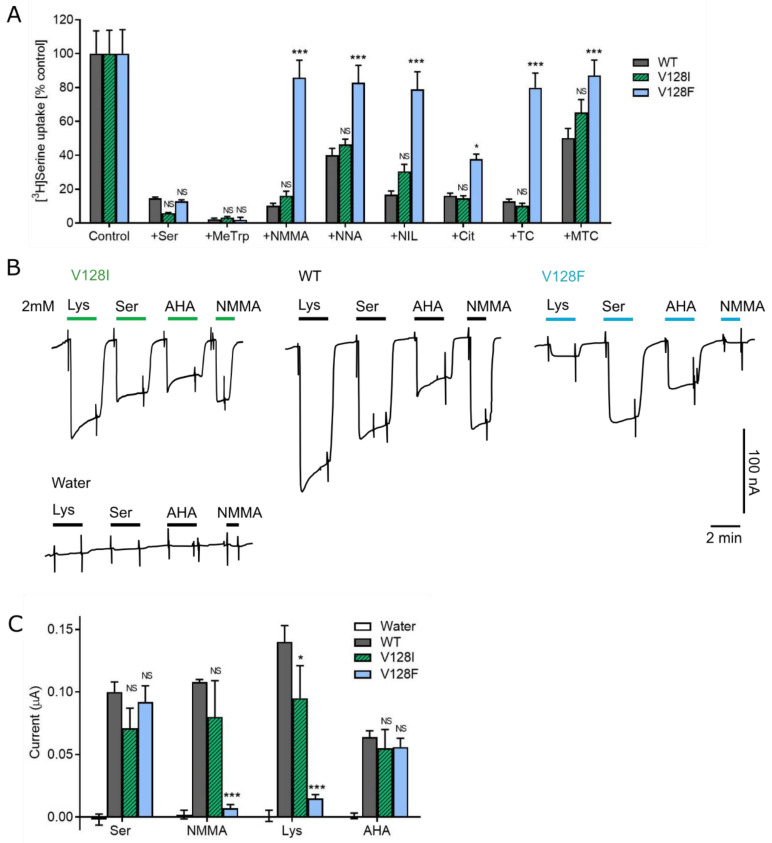
NOS inhibitors are not transported by the SLC6A14-V128F mutant. (**A**) Serine (10 µM) uptake in the absence (control) and presence of various amino acid analogues and the SLC6A14 inhibitor MeTrp in SLC6A14 wild-type (WT) and V128I and V128F mutants. Compounds are: MeTrp, α-methyl-dl-tryptophan; NMMA, *N*^G^-monomethyl-l-arginine; NNA, *N*^G^-nitro-l-arginine; NIL, *N*^6^-(1-iminoethyl)-l-lysine; Cit, l-citrulline; TC, l-thiocitrulline; MTC, S-methyl-l-thiocitrulline. All compounds were used at 2.5 mM except NIL which was used at 5 mM (due to its reported lower affinity for SLC6A14 [15]. SLC6A14-specific transport is shown (i.e., after the subtraction of uptake into water-injected oocytes, measured under identical conditions)). n = 18–20. ***, *p* < 0.001; *, *p* < 0.05; NS, *p* > 0.05 vs. WT. (**B**) Example traces showing transporter-associated inward current induced by various amino acids and analogues (all 2 mM) in SLC6A14-WT, V128I and V128F mutants, and water-injected oocytes as a control. AHA, 2-aminoheptanoic acid. (**C**) Mean inward currents induced by 2 mM of each compound [as shown in (**B**)] after 120 s of superfusion. n = 3–5. ***, *p* < 0.001; *, *p* < 0.05; NS, *p* > 0.05 vs. WT.

As well as accepting 18 of the 20 proteinogenic amino acids, SLC6A14 can transport several drugs and prodrugs [6,16,17] including arginine, lysine and citrulline analogues that act as NOS inhibitors such as *N*^G^-monomethyl-l-arginine (NMMA, tilarginine) [15]. Non-transported inhibitors of SLC6A14 such as α-methyl-dl-tryptophan (abbreviated to MeTrp in Figure 4A) have been shown to reduce cancer cell proliferation (for review, see [6]). The inhibition of wild-type SLC6A14 by α-methyl-dl-tryptophan was maintained in both the V128I and V128F mutants (Figure 4A). This is consistent with neither mutation preventing the binding of the aromatic amino acid substrate phenylalanine (Figure 3). The access of a range of cationic NOS inhibitors to the binding pocket of SLC6A14 and its two mutants was tested (Figure 4). All NOS inhibitors tested showed a reduction (or abolition) in their ability to inhibit [^3^H]serine uptake via SLC6A14-V128F compared to wild-type SLC6A14 or SLC6A14-V128I (Figure 4A). Similarly, superfusion of NMMA (2 mM) produced only 6.5% of the current it elicited in wild-type SLC6A14 (Figure 4B,C). Therefore, a multitude of different cationic structures, including NOS inhibitors such as NMMA, exhibit reduced access to the SLC6A14 binding site in the V128F mutant. In contrast, the bulky, dipolar, aromatic α-methyl-dl-tryptophan inhibitor continues to bind with high affinity.

Lysine and arginine differ from other proteinogenic amino acids not only because of the cationic nature of their side chains but also because they possess the longest side chains. To differentiate between which of these properties is influencing the ability of such cationic amino acids to access the SLC6A14-V128F binding pocket, lysine-induced currents were compared to those elicited by the dipolar amino acid derivative 2-aminoheptanoic acid (AHA) (Figure 4B,C). AHA and lysine have side chains that are of similar length but differ in that the amino group, found in the zeta position in the lysine side chain, is replaced by an uncharged methyl group in AHA. Lysine current was reduced by 89% from 140 ± 13 µA in wild-type to 15 ± 3 µA in SLC6A14-V128F (*p* < 0.001) consistent with the uptake results in Figure 3 whereas AHA current was not significantly different between the wild-type and V128F mutant (being 64 ± 5 µA in wild-type versus 56 ± 7 µA in SLC6A14-V128F, *p* > 0.05). Therefore, amino acids and derivatives with long, uncharged side chains are tolerated in SLC6A14-V128F whereas amino acids and derivatives carrying a positive charge on their side chain are not (Figure 4).

## 4. Discussion

SLC6A14 is unique among mammalian amino acid transporters in its ability to transport cationic as well as all dipolar amino acids [6,7,8,13,14]. SLC6A14 has been shown to be a major arginine uptake mechanism in both colon and lung epithelium which in turn modulates nitric oxide levels and nitric oxide signalling to downstream effectors such as the CFTR channel [25]. In some pathophysiological conditions including certain cancer types (colorectal, cervical, estrogen receptor positive breast, pancreatic) [28,29,30,31] the high demand for supply of amino acids to drive growth and proliferation is accomplished partly via increased expression of SLC6A14.

Even though the SLC6A14 transporter possesses a fascinating breadth of substrates, to our knowledge, there have been no investigations of the relationship between binding pocket structure and function in the SLC6A14 transporter thus far. In contrast, several of the other members of the SLC6 family, particularly those involved in reuptake of neurotransmitters, have been at the epicentre of transporter structure–function research for decades (examples include [62,63,64,65,66]). Re-evaluation of extensive functional studies [62,63,64,65,66] is enabled following the revelation of the structures of the prokaryote amino acid transporters LeuT [50,61] and MhsT [57], along with eukaryote structures of the *Drosophila* dopamine transporter dDAT [53], and human SLC transporters for serotonin (SLC6A4 or SERT) [54], amino acids (SLC6A19 or B^0^AT1; SLC6A9 or GlyT1) [51,52] and, most recently, GABA (SLC6A1 or GAT1) [67].

The LeuT structure can be considered as archetypal for transporters from 14 of the 18 transporter families within the APC superfamily [5,37] (including the 8 SLC families highlighted in Figure 1). However, there is, generally, no simple relationship between phylogeny and function alone with APC superfamily transporters varying greatly in the nature of their substrates as well as their mechanism of action (symport, antiport, uniport) emphasising the importance of coordinated investigations of sequence, structure and function [37]. Thus, subtle variation in the substrate binding pocket determines substrate specificity and affinity, providing each transporter type with a unique and identifiable set of functional characteristics [37]. 

Consistent with the earlier functional studies, examination of the occluded, substrate-bound, LeuT structure identified several positions within the LeuT-fold that were important in shaping the binding pocket [50] including residues in certain positions in TM3, 6 and 8 [50,61]. This included a Val residue (V104) in TM3 which occupied a deep position within this hydrophobic pocket [50,61]. Across the wider NSS family, the TM3 residue occupying that equivalent position in prokaryote and eukaryote transporters is generally one of the branched-chain amino acids Val, Leu or Ile (Figure 2) [68]. Functional studies in the SLC6 monoamine transporters SLC6A4 (SERT) and SLC6A3 (DAT) and the creatine transporter SLC6A8 (CreaT) [62,63,64,65,66] had shown that this position (I172 in SLC6A4, V152 in SLC6A3, C144 in SLC6A8) (Supplemental Figure S2) was in close proximity to the binding site and that alteration of the residue at that site influenced substrate and inhibitor selectivity. In the crystal structures of human SLC6A4 and *Drosophila* dDAT the residues occupying the equivalent position partly define the areas of the binding pockets associated with both substrate and antidepressant binding (Supplemental Figure S2) [53,54,69].

Moreover, in another LeuT-fold transporter family, the AAAP family (see Figure 1), the nature of the amino acid occupying the position equivalent to LeuT V104 appears to have a crucial role in determining substrate specificity in both vertebrate and invertebrate amino acid transporters [37,60]. The size of the amino acid residue side chain effectively titrates the space available within the binding pocket and thus determines accessibility of the amino acid substrate side chain [37]. Thus, the mammalian amino acid transporter SLC36A2 (PAT2), which is responsible for amino acid reabsorption from renal filtrate, has a large Phe residue at this position and so is limited to transport of amino acids with small side chains (glycine, alanine, proline) [37,41]. Introduction of a residue with a smaller side chain at that position broadens the SLC36A2 substrate specificity to include amino acids with longer side chains [37]. In contrast, the System N type transporter SLC38A5 (SNAT5), which is involved in amino acid transport in hepatocytes and astrocytes, has a relatively small amino acid (Ala) at the equivalent position to LeuT V104, and can transport amino acid substrates with relatively long side chains. Introduction of an amino acid residue with a longer side chain at that position alters substrate specificity and SLC38A5 changes in preference to transport small amino acids [37]. This knowledge has enabled prediction, testing and confirmation of the amino acid substrate selectivity in other members of the broader AAAP transporter family, *D. melanogaster* amino acid transporter CG1139 and the *Acyrthosiphon pisum* transporter ApNEAAT1, where substrate selectivity is determined by the available volume of space within that region of the transporter binding pocket [37,60]. Similarly, for example, in human SLC7 heteromeric amino acid transporters (HATs) from the APC family (2.A.3) (Figure 1A) the residue occupying the equivalent position is important in determining substrate selectivity in SLC7A5 (LAT1) and SLC7A8 (LAT2) [70,71].

Therefore, the earlier functional observations in the SLC6 family in members that transport monoamines or creatine [62,63,64,65,66], along with structural and functional observations in LeuT, SLC6A4 and dDAT [50,53,54,61,69], and functional and mutational studies in the more distantly related amino acid transporters from the AAAP family [37,60], led to this investigation of the LeuT V104 equivalent position in an amino acid transporter within the SLC6 family. The motivation for this was two-fold. Firstly, most prokaryote and eukaryote members of the NSS family [68], including most mammalian SLC6 transporters, show great unevenness in substrate specificity but little variability at the TM3 position having one of the branched-chain amino acids Val, Leu or Ile (Figure 2). Secondly, the nature of the substrate specificity even within a subgroup of the SLC6 amino acid transporters (Figure 2) varies enormously from high specificity (SLC6A5, SLC6A7, SLC6A9) to the great breadth of substrates transported by SLC6A14 [3]. Therefore, what role might the equivalent position to LeuT V104 play in determining substrate transport in SLC6A14 which can transport disparate compounds with relatively similar affinity (showing little preference for small, long, bulky, hydrophobic, polar or charged side-chains)?

A series of SLC6A14 mutants were investigated where the Val residue at position 128 (V128) was systematically increased in size [72] from Val (139.0 Å^3^) to Ile (163.9 Å^3^) to Phe (191.9 Å^3^) (Figure 3 and Figure 4). The V128I mutation had little overall effect on substrate specificity with all amino acids and derivatives investigated (aromatic, aliphatic, dipolar, dibasic) being transported (Figure 3). However, there was a notable five-fold improvement in the affinity (decrease in K_m_) for the small amino acid serine in the V128I mutant presumably due to smaller amino acids having a better fit within the V128I binding pocket. Similar observations have been noted previously in the SLC38A5 transporter with a marked improvement in access of the small amino acid alanine following mutation of the SLC38A5 A138 residue (90.0 Å^3^) to either of the larger amino acids Thr (124.7 Å^3^) or Ile [37].

In the SLC6A14-V128F mutant, the dipolar amino acids, of all sizes, appear to be transported similarly to that in the wild-type carrier (Figure 3 and Figure 4). This is somewhat surprising given the size of the substitution and suggests that the V128F binding pocket is still large enough, and flexible enough, to accommodate a broad range of compounds. The improved affinity for serine, observed in SLC6A14-V128I, was lost in the V128F mutant, presumably due to the larger Phe causing some additional changes in the shape and volume of the binding pocket. However, the striking difference in the SLC6A14-V128F mutant, versus either the wild-type transporter or the V128I mutant, was the almost complete loss of transport of the cationic amino acids arginine and lysine and related cationic NOS inhibitors (Figure 3 and Figure 4). Lysine and arginine not only have terminal charged moieties on their side chain but also have the longest side chains of any amino acid. Comparing transport of lysine to AHA (Figure 4) showed that it is the positively charged side chain, rather than length, that is the key characteristic causing differential recognition in SLC6A14-V128F compared to wild type. This is also consistent with the binding of large aromatic compounds such as phenylalanine and the inhibitor α-methyl-dl-tryptophan being maintained in the V128F mutant (Figure 3 and Figure 4). 

**Figure 5:**
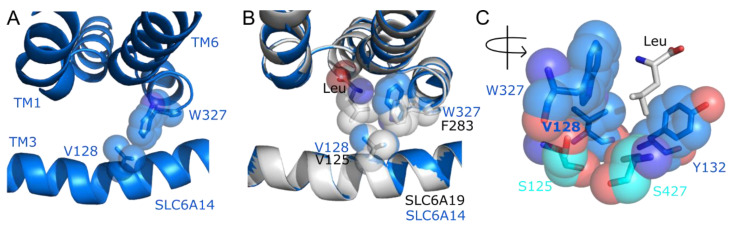
SLC6A14 V128 is in close proximity to a “sensor residue” position in TM6 of NSS transporters suggesting V128F could disrupt cation-π interactions between TM6 and cationic substrates or reduce flexibility of the substrate binding pocket. (**A**) Predicted structure of human SLC6A14 taken from the AlphaFold Protein Structure Database. Only sections of TM1, TM3 and TM6 are shown for clarity. V128 in TM3 and W327 in TM6 are shown as spheres and sticks. (**B**) Homology model of SLC6A14 (blue) created using, and overlaid on to, the crystal structure of SLC6A19 (grey, PDB: 6M17). The SLC6A19 substrate leucine and named residues (grey or blue) are shown as spheres and sticks. (**C**) Homology model of SLC6A14 created using the crystal structure of SLC6A19 (6M17). The image is rotated around the vertical axis compared to (**A**,**B**). Residues predicted to line the deep part of the binding pocket are shown as sticks and spheres with S427 (TM8) and S125 (TM3), which appear to form a polar cleft, highlighted in cyan. Other residues are V128 (TM3), the highly conserved gating residue Y132 (TM3), and W327 (TM6). To aid visualisation of the predicted binding pocket the leucine bound to the 6M17 crystal structure was included as for (**B**).

Therefore, unlike observations in the AAAP family, V128 in SLC6A14 plays a key role in determining substrate specificity in SLC6A14 (Figure 3 and Figure 4) perhaps not through an effect on binding pocket volume but rather due to a steric influence over the electrostatic compatibility between substrate and binding pocket. The V128F mutant could cause either the side chains of the cationic substrates to adopt different conformers and/or preclude the substrate side chain from a region of the binding pocket normally accessible within the wild-type SLC6A14. The modelling of SLC6A14 on known related structures (Figure 2 and Figure 5) suggests that V128 in SLC6A14 sits adjacent to two residues, one aromatic (TM6) and one polar (TM8), that occupy positions known to be important in substrate binding in LeuT-fold transporters (Figure 5) [50,53,66,69,73,74,75]. 

Firstly, the aromatic W327 in SLC6A14 is at the centre of the flexible, unwound region in TM6. In the SLC6 family, and LeuT, this residue is most commonly a Phe (F259 in LeuT). Figure 5 shows that threading SLC6A14 onto either LeuT or SLC6A19 places W327 adjacent to V128 where W327 lines the side and deep regions of the binding pocket. Cationic substrates could form cation-π interactions with the W327 side-chain indole. In SLC6A14, introduction of V128F might displace W327 and prevent potential cation-π interactions. There is excellent evidence that this TM6 position plays a key role in substrate binding in other NSS transporters. Two series of crystal structures showing different amino acid substrates bound to either LeuT [61] or the bacterial NSS transporter MhsT [76], show that this TM6 residue (F259 and M236 in LeuT and MhsT, respectively) moves or rotates to allow best fit of larger or smaller substrates. As such, both have been described as volumetric sensors [61,76,77] adapting binding site fit and volume within the hydrophobic environment. The calculated orientation of W327 in SLC6A14 varies between homology models made using different NSS family crystal structures. However, in models based on LeuT (2A65) or SLC6A19 (6M17), along with the AlphaFold prediction (Figure 2 and Figure 5A–C), the indole of W327 consistently projects towards V128, perpendicular to the plane of each crystal-bound substrate (Figure 5). Interestingly, the closely-related glycine transporters SLC6A9 and SLC6A5 are the only other SLC6 proteins to have a Trp in this position in the unwound region of TM6. However, the recently resolved crystal structure of inhibitor-bound SLC6A9 [52] places the equivalent Trp (W376) in a different orientation, from that predicted in SLC6A14, where the indole side chain in SLC6A9 sits above the V128 equivalent residue (I192 in SLC6A9) in TM3 and appears to preclude access to the latter from within the binding pocket (Supplemental Figure S4) [52]. This different orientation of the TM6 Trp residue between the broad scope transporter SLC6A14 (Figure 5) compared to SLC6A9 (Supplemental Figure S4) may provide a partial explanation for the highly restricted substrate specificity of the GlyT transporters SLC6A9 and SLC6A5 compared to the promiscuity of SLC6A14. Indeed, introducing a TM6 Trp to Phe mutation in the other glycine-selective transporter SLC6A5, reduces affinity for glycine but broadens substrate specificity to include many other dipolar amino acids including alanine, leucine and phenylalanine [78] suggesting that the Trp residue might normally restrict access to the deeper region of the binding pocket. Introducing the I192A mutation in SLC6A9 leads to loss of binding of the specific inhibitor Cmpd1 despite no direct contact being observed between the TM3 residue and the inhibitor [52]. SLC6A9 I192 reduces the rotational freedom of W376 in TM6. This confirms that mutating this position in TM3 influences the TM6 position in a transporter closely related to SLC6A14 [52].

Secondly, the modelling in Figure 5 suggests that SLC6A14 V128 sits adjacent to a polar Ser residue S427 in TM8 and a second Ser residue S125 in TM3. The two polar residues appear to form a polar cleft with their side chains, deep in the binding pocket (Figure 5C). This cleft may also include the polar side chain of T129 (a Thr at this position being unique among the SLC6 family) (Figure 2B). Introducing V128F could perhaps disrupt access of the substrate side chain to this subsite and, therefore, prevent the side chain nitrogens interacting with this polar cleft. Like the TM6 residue described above, the TM8 position described here is important in determining substrate specificity in other transporters. The SLC6A14 S427 equivalent position in TM8 of LeuT (I359) has been shown to rotate in response to varying substrate size [61,77]. In addition, I359Q in LeuT converts tryptophan from a non-transported inhibitor (which wedges LeuT in the outward-open conformation) to a transported substrate [75]. In more distantly related LeuT-fold APC superfamily transporters such as the APC family (2.A.3) [5,37], which includes the mammalian SLC7 family of CAT (cationic amino acid) transporters (Figure 1), the size of the residue in the TM8 position described above also appears to be an important determinant of binding pocket volume. Unlike the human SLC7 CAT transporters, which are highly selective for the cationic amino acids arginine and lysine, the bacterial transporter GkApcT is a broad specificity transporter with preference for small hydrophobic and polar amino acids [79]. Mutation of the TM8 position in GkApcT from the relatively large Met to the smaller Ser (M321S), to imitate the Ser found in the human CAT transporters, created a high-affinity arginine transporter, which is proposed as being due to removal of the steric constraints imposed on the binding pocket by the larger Met in wild-type GkApcT [79].

The TM3, 6 and 8 positions are also important in biogenic amine transporters of the SLC6 family [53,69]. For example, the equivalent positions in TM3, 6 and 8 all sculpt the binding pocket in dDAT and contribute to the relative affinity of the carrier for dopamine, cocaine and various antidepressants [53,69].

Overall, the SLC6A14 transporter has the LeuT architecture typical of the NSS family but which, in addition to the transport of dipolar amino acids, has the additional capacity for cationic amino acid transport. Interestingly, some of the features of the SLC6A14 binding pocket described here are reminiscent of those found in the resolved structure of the APC family (2.A.3) arginine transporter AdiC (2.A.3.2.5). The cationic substrates of SLC6A14 could form cation-π interactions with the W327 side-chain indole in a manner analogous to the guanidinium-Trp interaction between bound arginine and W293 (TM8) of AdiC [73]. In addition, in AdiC, the nitrogen atoms of the guanidinium group of the arginine substrate are close to the side-chain oxygen atoms of S357 (TM10) and N101 (TM3) [73,74] which is a role proposed for TM3 and TM8 Ser residues in SLC6A14.

In conclusion, to understand what any membrane transporter does in physiological terms, we first need to understand what it transports and why it is selective for one substrate over another. Such knowledge will provide the foundation for accurate in silico methods to enable predictive modelling of transporter function, drug delivery and transport, and the mechanism of action of novel pharmaceutical treatments for pathophysiological conditions and disease states. The LeuT-fold architecture has evolved to produce a huge number of amino acid transporters with a myriad of unique substrate specificities which allows these transporters to fulfil a diverse range of physiological roles. The TM3 residue described here is a key component of the binding pocket in many such transporters. Here, we show that substituting V128 in the broad-specificity SLC6A14 amino acid transporter modulates substrate fit and, specifically, alters cationic amino acid and cationic NOS inhibitor transport.

## Data Availability

Data presented in this investigation are available within the article and supplementary material. Alignments used in phylogenetic analyses are available at 10.6084/m9.figshare.20182088.

## Supplementary Materials

The following supporting information can be downloaded at: https://www.mdpi.com/article/10.3390/biom12101404/s1, Figure S1: Amino acid transporters within multiple families of the APC superfamily and the Neurotransmitter:Sodium Symporter family [80]; Figure S2: V128 occupies a key position, towards the bottom of the binding pocket, in SLC6A14 and in other SLC6 transporters; Figure S3: V128F in TM3 reduces transport of the dibasic amino acid arginine via SLC6A14 whereas V128I increases affinity for the small dipolar amino acid serine; Figure S4: The orientation of W376 in the SLC6A9 (GlyT1) transporter differs from that predicted for W327 in SLC6A14 and “blocks” the TM3 residue I192 from the binding pocket [52].
