# Supplementary material for: Reshaping the Binding Pocket of the Neurotransmitter:Solute Symporter (NSS) Family Transporter SLC6A14 (ATB0,+) Selectively Reduces Access for Cationic Amino Acids and Derivatives
Source: Biomolecules. 2022 Oct 1;12(10):1404. doi: 10.3390/biom12101404 (PMC9599917; doi:10.3390/biom12101404)
Supplement: Supplementary file 1 [file biomolecules-12-01404-s001.zip › biomolecules-1823452-supplementary.pdf]

Supplemental Information for:

Reshaping the Binding Pocket of the Neurotransmitter:Solute Symporter (NSS) Family Transporter SLC6A14 ($ATB^{0,+}$) Selectively Reduces Access for Cationic Amino Acids and Derivatives.

Catriona M. H. Anderson[1,*], Noel Edwards[2], Andrew K. Watson[2], Mike Althaus[1,3] and David T. Thwaites[2,*]

[1] School of Natural & Environmental Sciences, Faculty of Science, Engineering & Agriculture, Newcastle University, Newcastle upon Tyne, NE1 7RU, UK;

[2] Biosciences Institute, Faculty of Medical Sciences, Framlington Place, Newcastle University, Newcastle upon Tyne, NE2 4HH, UK;

[3] Department of Natural Sciences / Institute for Functional Gene Analytics, Bonn- Rhein-Sieg University of Applied Sciences, 53359 Rheinbach, Germany;

* Correspondence: catriona.anderson@ncl.ac.uk (C.M.H.A.); d.t.thwaites@ncl.ac.uk (D.T.T.)

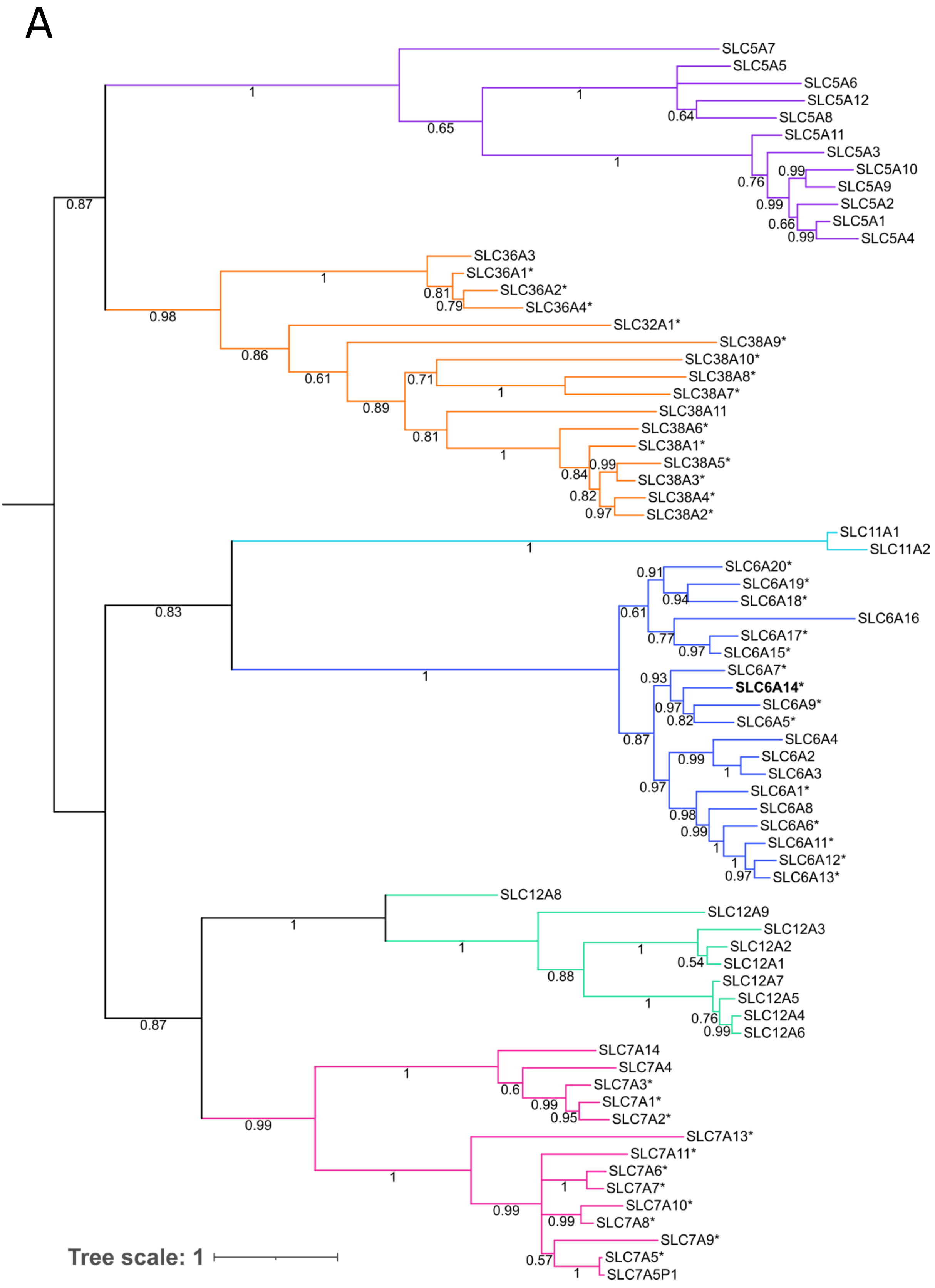
A
SLC5A7
SLC5A5
SLC5A6
SLC5A12
SLC5A8
SLC5A11
SLC5A3
SLC5A10
SLC5A9
SLC5A2
SLC5A1
SLC5A4
SLC36A3
SLC36A1*
SLC36A2*
SLC36A4*
SLC32A1*
SLC38A9*
SLC38A10*
SLC38A8*
SLC38A7*
SLC38A11
SLC38A6*
SLC38A1*
SLC38A5*
SLC38A3*
SLC38A4*
SLC38A2*
SLC11A1
SLC11A2
SLC6A20*
SLC6A19*
SLC6A18*
SLC6A16
SLC6A17*
SLC6A15*
SLC6A7*
SLC6A14*
SLC6A9*
SLC6A5*
SLC6A4
SLC6A2
SLC6A3
SLC6A1*
SLC6A8
SLC6A6*
SLC6A11*
SLC6A12*
SLC6A13*
SLC12A8
SLC12A9
SLC12A3
SLC12A2
SLC12A1
SLC12A7
SLC12A5
SLC12A4
SLC12A6
SLC7A14
SLC7A4
SLC7A3*
SLC7A1*
SLC7A2*
SLC7A13*
SLC7A11*
SLC7A6*
SLC7A7*
SLC7A10*
SLC7A8*
SLC7A9*
SLC7A5*
SLC7A5P1
Tree scale: 1

B

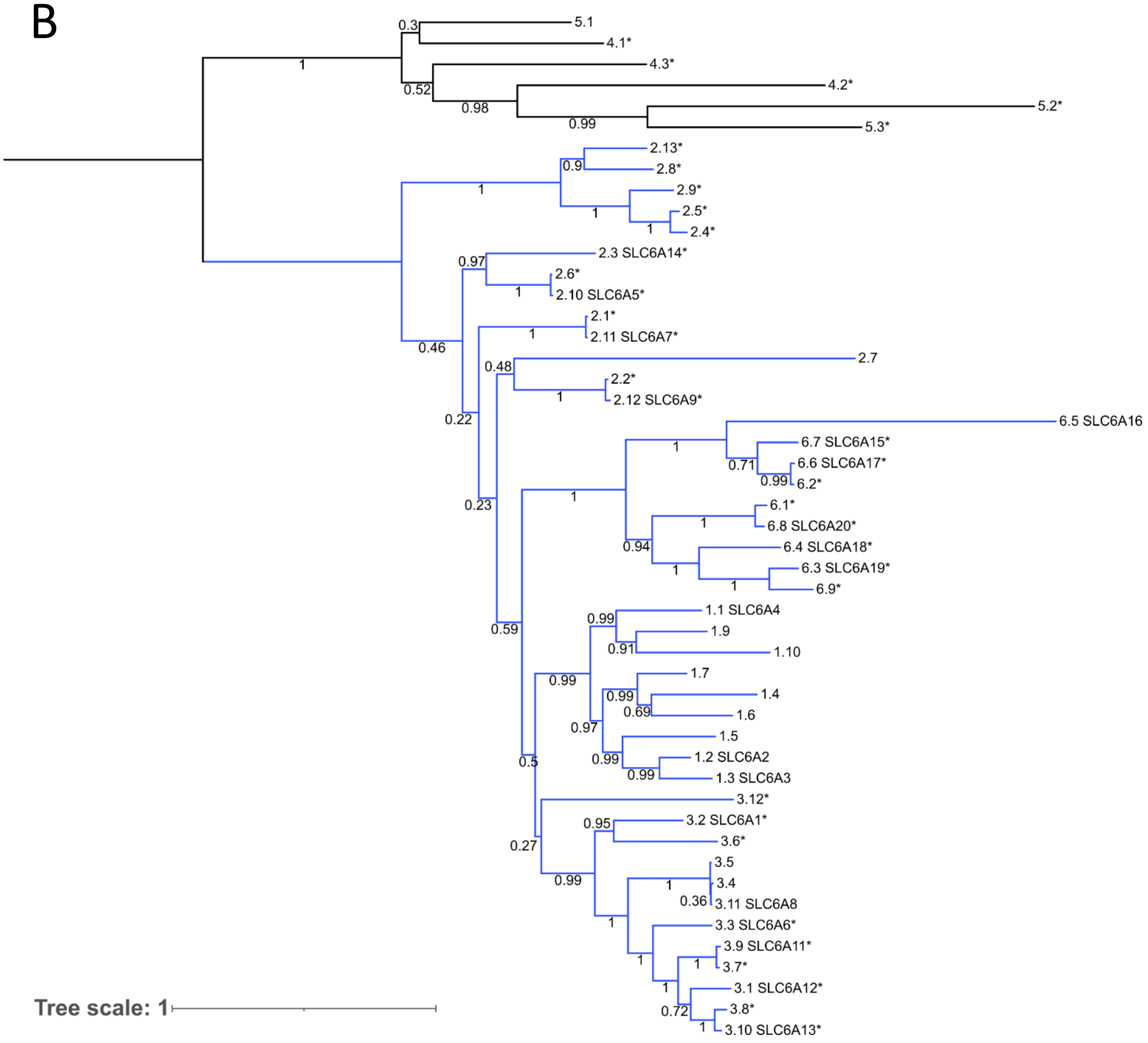

Figure S1. Amino acid transporters within multiple families of the APC superfamily and the Neurotransmitter:Sodium Symporter family. (**A**) Phylogenetic analysis of human APC superfamily proteins as described in Fig. 1A. The tree was inferred using Phylobayes and the C60 model, and midpoint rooted in iTol [Letunic and Bork, 2021]. (**B**) Phylogenetic analysis of the NSS (2.A.22) family as described in Fig. 1B. The tree was inferred using Phylobayes and the C20 model and midpoint rooted in iTol. Asterisks indicate proteins with known amino acid transport function. Values on branches are posterior probabilities, indicative of the level of support for that branch.

Reference:
Letunic, I. and Bork, P. Interactive Tree Of Life (iTOL) v5: an online tool for phylogenetic tree display and annotation. *Nucleic Acids Res,* **2021**, *49*, W293-W296. doi: 10.1093/nar/gkab301

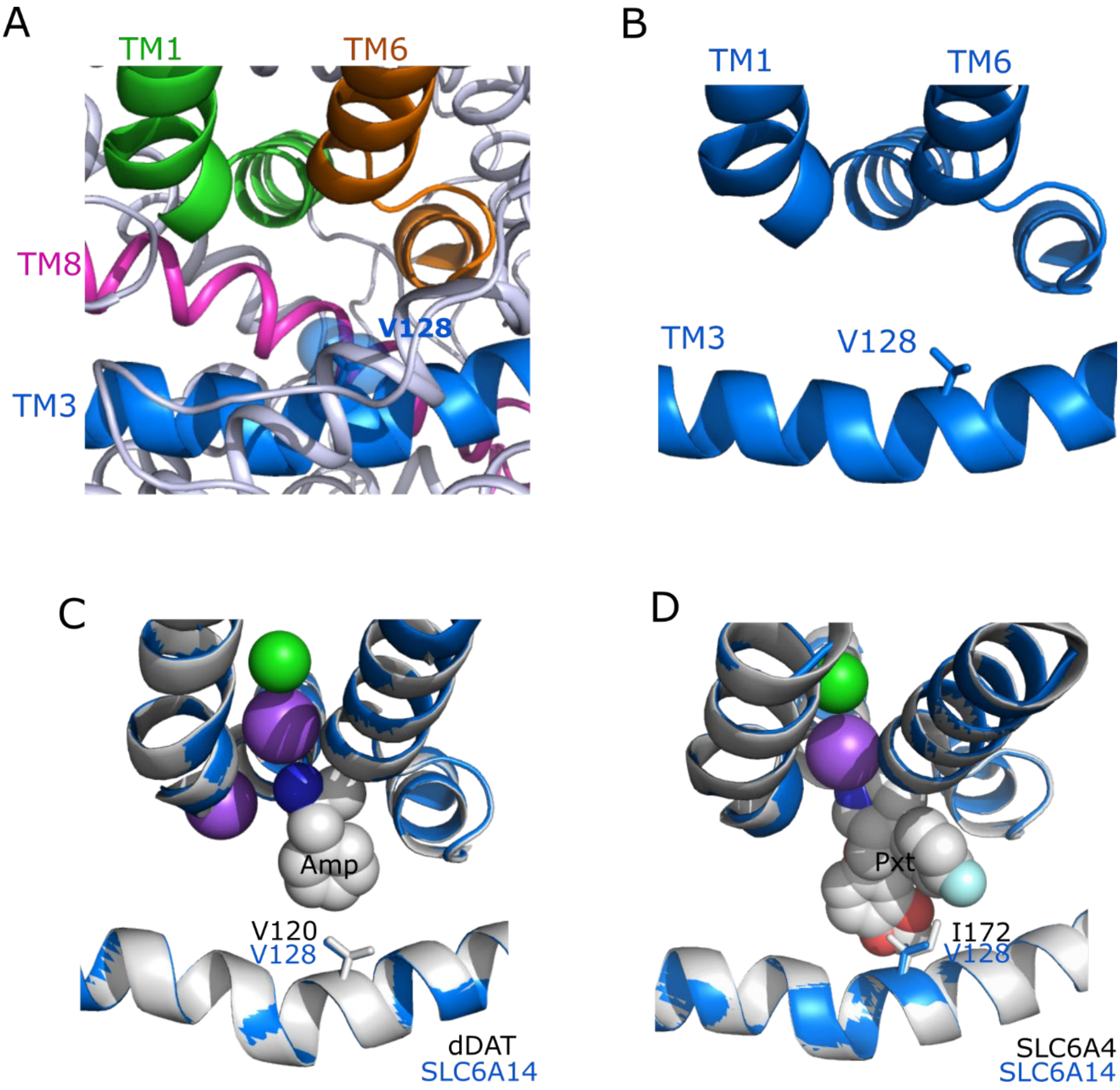

Figure S2. V128 occupies a key position, towards the bottom of the binding pocket, in SLC6A14 and in other SLC6 transporters. (**A**) Predicted structure of human SLC6A14 taken from the AlphaFold Protein Structure Database. Protein is coloured as for Figure 2A whereby all residues are grey except TM1 (green), TM3 (blue), TM6 (orange) and TM8 (magenta) which line the binding pocket in many LeuT-fold transporters. V128 in TM3 is shown as blue spheres. (**B**) The same view of SLC6A14 generated by AlphaFold as shown in A but only showing TM1, TM3 and TM6 (all blue) and V128 as blue sticks. (**C**, **D**) Homology models of SLC6A14 created using dopamine transporter (dDAT) from *Drosophila melanogaster* [4XP9, with substrate D-amphetamine (Amp)] and human SLC6A4 (SERT) [5I6X, with competitive inhibitor paroxetine (Pxt)], respectively.

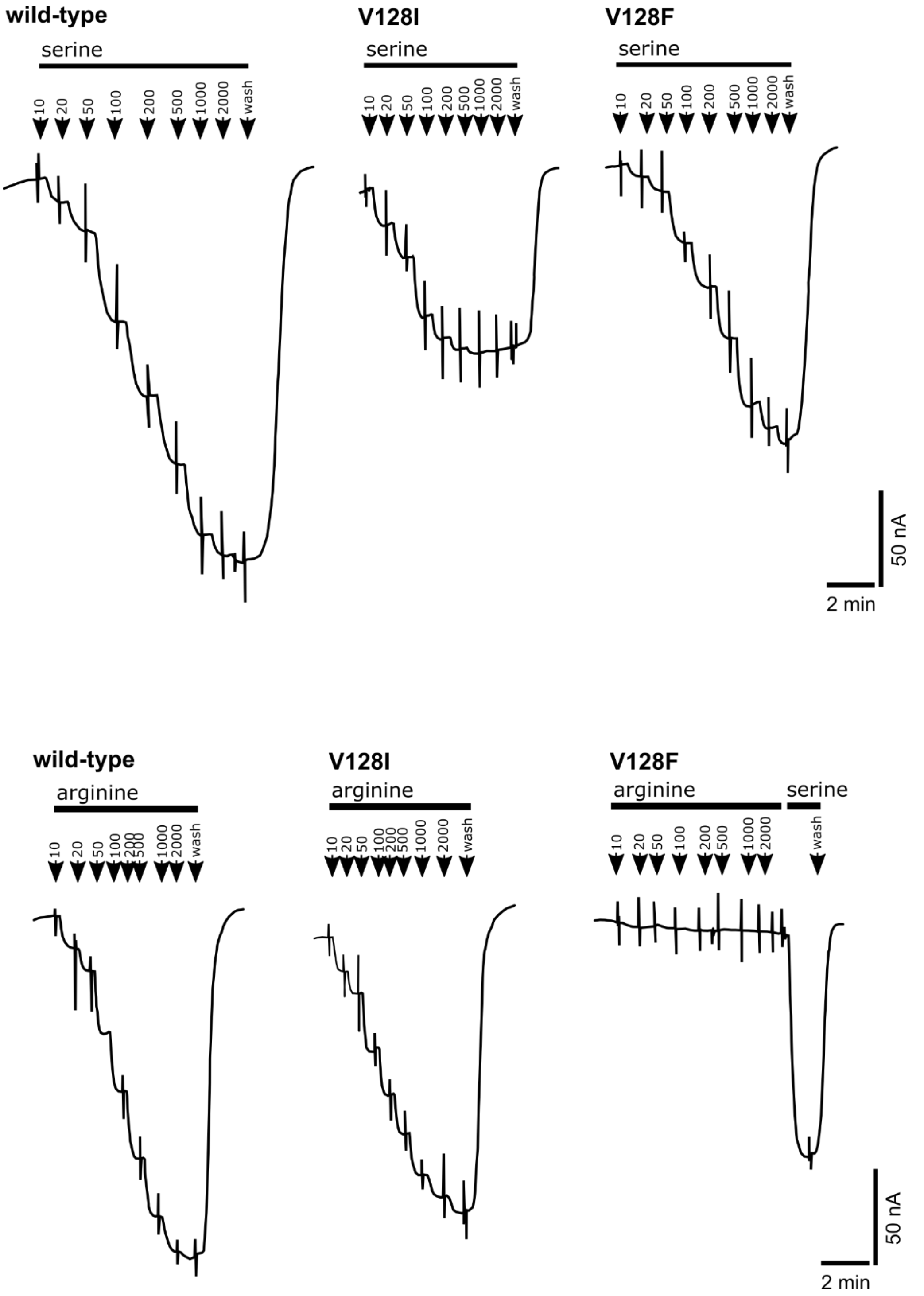

Figure S3. V128F in TM3 reduces transport of the dibasic amino acid arginine via SLC6A14 whereas V128I increases affinity for the small neutral amino acid serine. Example traces showing serine or arginine-associated inward current measured by TEVC in oocytes expressing SLC6A14 wild-type or V128I or V128F mutants. Increasing concentrations (10-2000 μM) of either amino acid were added sequentially. As no current was associated with arginine in V128F, 2 mM serine was used as a control. Mean data are shown in Figure 3E,F. Current associated with each concentration of amino acid was calculated 60 s after addition of amino acid. Current measured prior to amino acid addition (i.e. in superfusion solution) was subtracted to give SLC6A14-associated current.

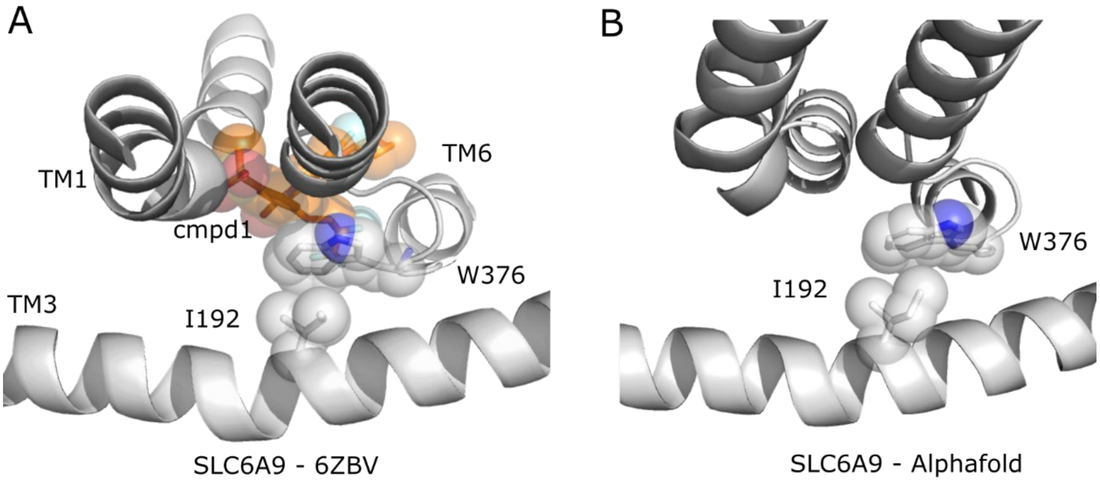

Figure S4. The orientation of W376 in the SLC6A9 (GlyT1) transporter differs from that predicted for W327 in SLC6A14 and "blocks" the TM3 residue I192 from the binding pocket. (**A**) Structure of SLC6A9 taken from Shahsavar et al. (2021) (6ZBV, inward open). (**B**) The predicted structure of SLC6A9 from the Alphafold Protein Structure Database. Sections of TM1, TM3 and TM6 are shown with I192 (TM3) and W376 (TM6) as grey sticks and spheres. The GlyT1 inhibitor Cmpd1, which wedges the transporter in the inward open confirmation, is shown as orange sticks and spheres.

Reference:
Shahsavar, A.; Stohler, P.; Bourenkov, G.; Zimmermann, I.; Siegrist, M.; Guba, W.; Pinard, E.; Sinning, S.; Seeger, M.A.; Schneider, T.R.; Dawson, R.J.P.; Nissen. P. Structural insights into the inhibition of glycine reuptake. *Nature*, **2021**, *591*, 677-681. doi: 10.1038/s41586-021-03274-z
